# Study on the Influence of Walnut Shell Coarse Particles on the Slurry Permeation and the Air Tightness of Filter Cake

**DOI:** 10.3390/ma17215186

**Published:** 2024-10-24

**Authors:** Qi Dong, Tao Liu, Yuan Wang, Sijin Liu, Letian Wen

**Affiliations:** 1 College of Water Conservancy and Hydropower Engineering, Hohai University, Nanjing 210024, China; dong0626qi@hhu.edu.cn (Q.D.);; 2 College of Civil Engineering and Transportation, Hohai University, Nanjing 210024, China; 3 China Railway 14th Bureau Group Co., Ltd., Jinan 250000, China; 4 China South-to-North Water Diversion Middle Route Co., Ltd., Beijing 100036, China

**Keywords:** slurry shield, coarse particle, walnut shells, slurry infiltration, filter cake, air tightness

## Abstract

Slurry shields rely on the formation of a compact filter cake to maintain excavation face stability and ensure construction safety. In strata with high permeability, significant slurry loss occurs, making filter cake formation and air tightness maintenance challenging. In this study, light organic walnut shell was selected as an additive coarse particle material for slurry. Slurries incorporating two types of coarse particles, sand and walnut shell, were prepared, and tests on slurry permeation and air tightness of the filter cake were conducted in three different strata. The results indicate that the addition of coarse particles effectively improves filter cake formation and air tightness in high-permeability strata. It is essential to use graded particles in highly permeable strata, with controlled maximum and minimum particle sizes. As the content of coarse particles increases, the air tightness of the filter cake initially increases and then decreases. Notably, the air tightness of filter cakes containing walnut shell is superior to those containing sand. Replacing sand with walnut shell as a slurry plugging material enhances filter cake quality in high-permeability strata. For highly permeable strata with a permeability coefficient greater than 1.0 × 10^−3^ m/s, an addition of 30 g/L to 40 g/L is recommended.

## 1. Introduction

The slurry shield method is widely applied in the construction of underwater tunnels [1,2]. The filter cake formed by the infiltration of slurry into the excavation face acts as the first barrier to maintain the stability of the excavation face and ensure construction safety, making it a focal point in related research [3,4]. The pressurized slurry in the excavation chamber infiltrates forward, with the solid phase in the slurry gradually filling the surface pores of the strata to form a slightly permeable filter cake. The slurry pressure acts on the excavation face through the filter cake in the form of surface force, balancing the external soil and water pressure of the strata [5]. Talmon et al. [6] proposed that the condition for filter cake formation is that the particle deposition rate is greater than the infiltration rate of the slurry into the strata. Kou et al. [7] identified three infiltration modes and the deposition patterns of slurry filter cake formation through extensive experiments, each corresponding to different types of infiltration curves. On this basis, many scholars have studied the impact of slurry properties [8,9] on the formation effectiveness of filter cakes and have attempted to enhance the quality of the filter cake by using various slurry additives [10,11,12,13].

In low-permeability strata, the expansion and cementation of small particles in pure bentonite slurry are sufficient to form a filter cake. However, in highly permeable strata (such as sand and gravel layers), the large pores of the strata cause significant loss of pure bentonite slurry, making filter cake formation difficult. Therefore, a certain amount of coarse particles must be added to the slurry to block the pores of the strata. The classical filtration model theoretically explains the deposition and growth of the filter cake [14]. Tien et al. have improved the conventional cake filtration model based on multiphase flow equations [15], and Herzig et al. [16] proposed the mechanism for particle clogging and retention based on this model. In practice, the formation of a high-quality filter cake requires good compatibility between the slurry particles and the strata. Traditional theories generally classify filter cake types based on the ratio of particle diameter in the slurry to the pore diameter of the strata. Common matching criteria include the ‘1/3, 1/7 rule’ and the ‘1/3, 1/14 rule’ [17]. Min et al. [18] proposed a formation compatibility criterion based on the average pore diameter of the strata and the d_85_ of the slurry. In the field of shield tunneling construction, the most commonly used coarse particles are quartz sand [19,20,21]. Lin et al. suggested that the slurry filtration process starts with the large particles, and sand-added slurry can significantly accelerate the formation of filter cakes in coarse strata [22]. Ma et al. proposed that the solid particle size of sands has a significant effect on the filter cake growth process [23]. However, due to its high specific gravity, quartz sand tends to settle at the bottom of the excavation chamber, leading to an uneven filter cake, which fails to meet the quality requirements for shield machine tunneling and chamber opening. In recent years, organic coarse particle materials such as natural nutshells [24,25], plant fibers [26,27], and eggshells [28,29], which are used as lost circulation materials in drilling slurry, have attracted attention and have been increasingly used in shield tunneling construction due to their better compatibility with the slurry [30].

Additionally, as the shield tunneling distance increases, the tunnel boring machine (TBM) often requires chamber inspections due to tool wear or encountering boulders [31]. To maintain the stability of the excavation face during pressurized chamber openings, the filter cake must possess a certain degree of air tightness, which imposes higher quality requirements on the formation of filter cakes [32,33]. The failure of the filter cake under certain air pressure conditions is mainly due to the ingress of air, which occupies the pores of the filter cake and causes a sharp decrease in its internal saturation [34,35,36,37]. Additionally, with increasing air pressure and its duration, the pore structure of the filter cake changes [38]. The commonly used evaluation indicators for the air tightness of the filter cake are the air tightness pressure value and air tightness time [39]. The pore structure, thickness of the filter cake, permeability coefficient of the strata [40], and the content of coarse particles [41] all affect the air tightness performance of the filter cake.

To address the issue of poor filter cake quality caused by significant settling of sand particles in traditional sand-containing slurry in highly permeable strata, lightweight organic walnut shells were selected as slurry plugging additives due to their good plugging effect and durability [42]. Comparative tests on slurry infiltration and the air tightness of the filter cake were conducted using both sand-containing and walnut shell slurries in three sand layers of different permeabilities. This study analyzed the influence and mechanisms of coarse particle type, particle size, particle content, and strata permeability coefficient on the formation and air tightness of filter cakes. The findings aim to provide guidance for the application of walnut shells in shield tunneling and pressurized maintenance operations in highly permeable strata.

## 2. Experimental Materials and Methods

### 2.1. Experimental Materials

#### 2.1.1. Strata Materials

To study the slurry infiltration and filter cake formation in highly permeable sand layers with different pore structures, three types of strata with varying permeability were prepared using well-graded natural river sand. These strata, ordered by increasing permeability coefficient, are S1 (grain size 0.5–1 mm), S2 (grain size 2–5 mm), and S3 (grain size 3–5 mm). The basic parameters of each strata are listed in Table 1. The average pore diameter of the strata is calculated based on the characteristic pore value proposed by Min et al. [18]. The permeability coefficients of the strata were measured using the constant head permeability test.

#### 2.1.2. Slurry Materials

The materials used for slurry preparation in the experiments mainly included purified water, sodium-based bentonite, carboxymethyl cellulose (CMC), walnut shell particles, and sand particles. The base slurry was prepared at a water-to-bentonite ratio of 1:8, with a certain mass fraction of CMC added as a thickening agent. Each group of slurry was formulated by adding different types and amounts of coarse particles to the base slurry. The sodium-based bentonite was sieved using a 200-mesh (0.075 mm) standard sieve (Huafeng Hardware Instruments Co., Ltd., Shaoxing, China) to remove impurities. The particle size of the coarse particles was determined based on the d_85_ criterion proposed by Min et al. [18] and the standard sieve apertures used in the field. Coarse particles were added as particle groups rather than single particle sizes. The experimental groups and basic properties of the slurries are shown in Table 2.

### 2.2. Test Apparatus

**Figure 1:**
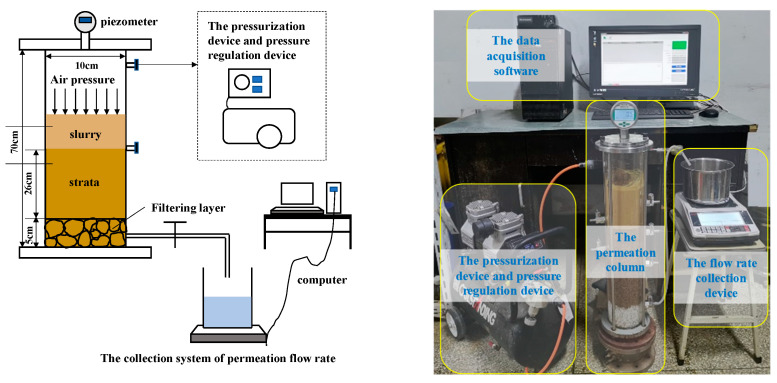
Test system of slurry permeation and filter cake air tightness.

The experiment utilized a self-made test system of slurry permeation and filter cake air tightness, comprising three main components: the pressurization system, the permeation column, and the collection system of permeation flow rate. The pressurization system consisted of a pressurization device (air compressor, Aotus Industry and Trade Co., Ltd., Taizhou, China) and a pressure regulation device (pressure regulator valve and bidirectional pressure gauge, Bowei Pressure Regulator Factory, Taizhou, China). The permeation column was made of organic glass with an inner diameter of 10 cm and a height of 70 cm, sealed at the top by a flange plate and connected to the flow rate collection device at the bottom via an open end. The permeation flow rate collection system included a high-precision balance, a filtrate collector, and a data acquisition software program (Anheng Flow Data Acquisition Software, and the version number is ACS-30), as shown in Figure 1.

### 2.3. Experimental Methods

Firstly, the strata filling process was initiated, with a bottom filter layer comprising white gravel with a particle size range of 6–8 mm and a height of 5 cm. The experimental strata consisted of well-graded natural river sand with a height of 26 cm. The dry density of the strata was strictly controlled to be the same for each test group. After filling the strata, the saturation process was conducted from bottom to top for 24 h. Once saturation was achieved, 1000 mL of prepared slurry was slowly injected. The slurry permeation test commenced after a 5 min settling period.

The slurry permeation test was conducted using a staged pressurization method, with pressures of 50 kPa, 100 kPa, 150 kPa, and 200 kPa applied successively, each maintained for 3 min. Throughout the experiment, the water outlet valve at the bottom of the permeation column remained open, and the data acquisition system automatically recorded the flow rate at 1 s intervals. After the test, the permeation column was slowly depressurized, and remaining slurry was drained by opening the valves on the side of the permeation column. The filter cake morphology was observed. The thickness and penetration distance of the filter cake were measured.

For the slurry groups that stable filter cakes formed during the permeation test, air tightness tests of the filter cakes were conducted. Each slurry group underwent two air tightness tests to determine the air tightness pressure value and air tightness time. After the formation of the filter cake, any remaining slurry in the permeation column was drained to allow direct air pressure on the filter cake surface. Air tightness pressure values were determined using staged pressurization. The pressure was incrementally increased by 30 kPa until filter cake failure occurred, with each pressure level maintained for 1.0 min. Air tightness time was measured under a constant pressure of 30 kPa until failure of the filter cake. Flow rate monitoring was conducted throughout the test, and the filter cake was removed after completion and the failure morphology was observed.

## 3. Results

### 3.1. Impact of Coarse Particle Materials on the Formation Characteristics of Filter Cake

#### 3.1.1. Influence of Coarse Particle Size on Filter Cake Formation

**Figure 2:**
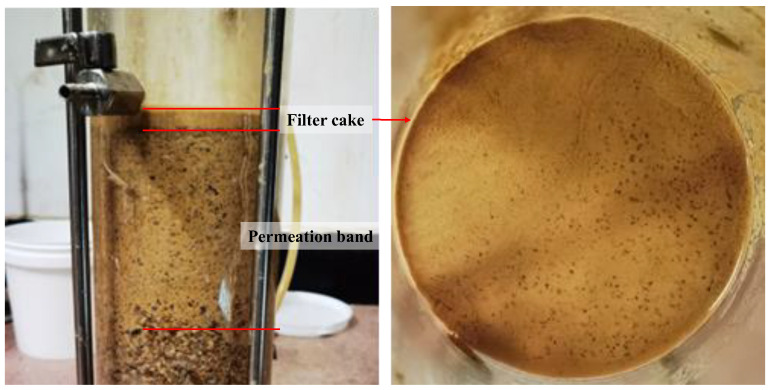
The combination type of permeation band and filter cake.

The types of low-permeability zones formed by the permeation of various experimental slurries are shown in Table 3. Two types of low-permeability zones were observed, which were the combination type of filter cake and permeation band, as shown in Figure 2, and penetrating-type permeation bands.

In all three strata, slurries without coarse particles failed to form a filter cake. According to the d_85_ matching criterion, the required particle sizes for filter cake formation in the S1, S2, and S3 strata should be 0.075–0.15 mm, 0.25–0.5 mm, and 0.5–1 mm, respectively. In the S1 and S2 strata, except for the slurry containing 10 g/L of 0.25–0.5 mm sand particles failing to form the filter cake in the S2 strata, all other slurries formed low-permeability zones characterized by the combination type of filter cake and permeation band. However, in the S3 strata, none of the slurries were able to form a filter cake. Therefore, while the particle sizes determined by the d_85_ criterion meet the requirements for filter cake formation in the relatively low-permeability S1 and S2 strata, they fail to meet the requirements for filter cake formation in the relatively high-permeability S3 strata.

**Figure 3:**
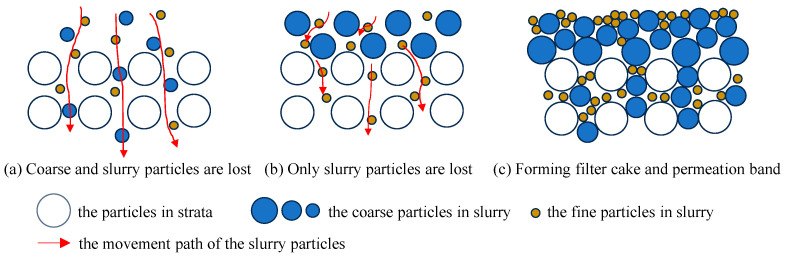
The bridging effect of coarse particles with different gradations in slurry.

To address this issue, additional permeation tests were conducted in the S3 strata using slurries supplemented with coarse particles in the particle size ranges of 0.25–0.5 mm, 0.5–1.0 mm, and 0.25–1.0 mm. The results revealed that slurries containing 0.25–0.5 mm coarse particles only formed permeation bands, with particles and slurry infiltrating the strata through its pores, as depicted in Figure 3a. Similarly, slurries containing 0.5–1.0 mm coarse particles also only formed permeation bands, with most coarse particles remaining on the surface of the strata, while fine particles in the slurry permeated through the pores formed by the accumulation of coarse particles, as illustrated in Figure 3b. However, slurries containing 0.25–1.0 mm coarse particles were able to generate a stable filter cake and permeation band combination, as shown in Figure 3c.

#### 3.1.2. Analysis of Permeation Flow in Filter Cake Formation

**Figure 4:**
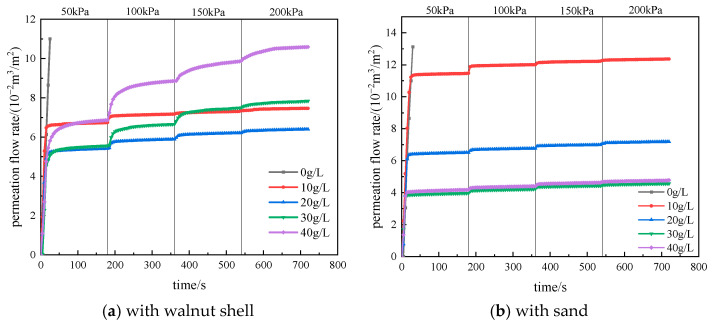
Infiltration flow rate curves of slurry in S3 stratums.

Under a certain pressure, the slurry infiltrates into the strata, with the liquid phase permeating into the strata while the solid phase accumulates on the surface to form a filter cake. The formation of the filter cake gradually reduces the permeability of the strata, hindering further infiltration of the liquid phase. Figure 4 depicts the variation in permeation flow during the formation of the filter cake in the slurry containing two types of coarse particles (using the S3 strata as an example).

**Figure 5:**
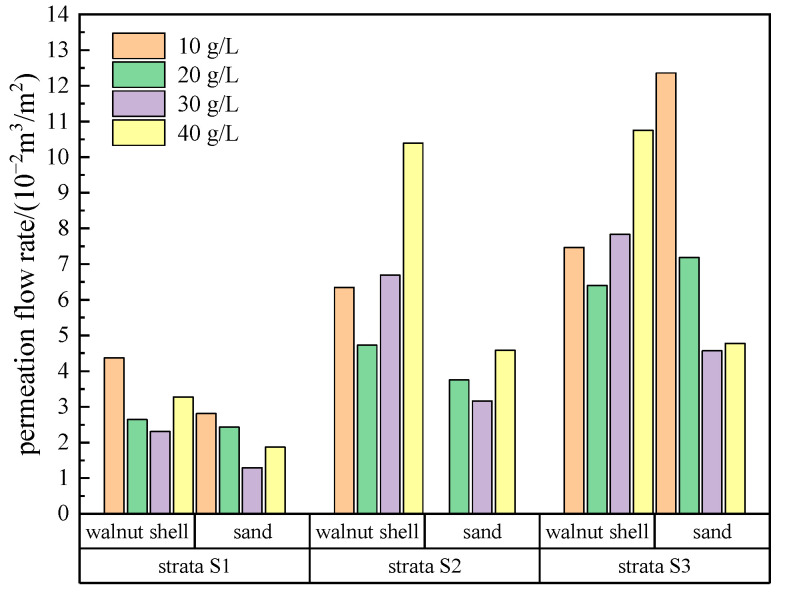
Infiltration flow volume of forming filter cake under varied permeability strata and coarse particle content.

After the application of pressure, the slurry permeation flow initially increases rapidly and then gradually stabilizes, indicating the formation of the filter cake. The pressure acts on the strata through the filter cake in the form of effective stress. In the experiment, each increase in pressure level results in a sudden change in flow rate, mainly due to filter cake compression and local breakthroughs, which then stabilizes as the filter cake is compacted or repaired. During the incremental pressurization process, the maximum flow rate is observed at the first pressure level, and subsequent pressure increments result in smaller flow rate changes, indicating the gradual densification of the filter cake. The statistical analysis of the permeation flow rates for each group of slurry is shown in Figure 5. Except for the slurry with 10 g/L of sand in the S2 strata, which did not form a filter cake, all other slurry groups formed stable filter cakes and permeation bands.

When slurry with the same type of coarse particles is permeating in strata with a fixed permeability coefficient, the permeation flow rate initially decreases and then increases with the increase in the amount of coarse particle addition, reaching a minimum value at a coarse particle content of 20 g/L or 30 g/L. Since this experiment is for vertical permeation, the permeation flow rate of slurry containing sand particles is slightly lower than that of slurry containing walnut shell particles. This is because sand particles have a higher density and can reach the strata surface more quickly under the action of gravity to block it, resulting in a shorter filter cake formation time and lower permeation flow rate. However, in actual construction, the permeation is lateral, and the density difference between the base slurry and sand particles can cause particle sedimentation, resulting in uneven filter cake quality. Additionally, as the permeability coefficient of the strata increases, the permeation flow rate of slurry containing both types of particles also increases.

#### 3.1.3. Permeability Analysis of Filter Cakes

The permeability coefficient of the filter cake is one of the key indicators to measure the quality of the filter cake. The process of slurry infiltration can be simplified into a one-dimensional seepage model. According to Equation (1), the permeability coefficient of the filter cake can be expressed as

(1) $$K=\frac{qL}{Ah}$$

where *K* represents the permeability coefficient of the filter cake, measured in m/s; *q* denotes the flux rate per unit time, measured in m^3^/s; *A* stands for the cross-sectional area of the sample, measured in m^2^, which corresponds to the cross-sectional area of the permeation column in the experiment, taken as 7.85 × 10^−3^ m^2^; *h* represents the water level difference at both ends of the sample, measured in m, which is taken as the final pressure difference during the slurry infiltration to form the filter cake in the experiment, specifically 20 m; and *L* stands for the length of the permeation path, which in the experiment is taken as the total thickness of the filter cake, measured in m.

The permeability coefficients of the filter cakes in each group are shown in Table 4. The permeability coefficients of the filter cakes formed by slurry infiltration in each group have reached the order of magnitude of 10^−7^ m/s or even smaller, effectively blocking the pores of the strata and forming low-permeability zones, meeting the requirements of shield tunneling construction.

When slurry with the same type of coarse particles permeates the same strata, the higher the amount of particle addition, the greater the permeability coefficient of the formed filter cake. This is because with more particles, the resulting filter cake becomes thicker and more porous, enhancing its compressibility. Consequently, during permeation, the increase in permeation flow rate due to the compression of the filter cake leads to longer permeation distances, resulting in a higher permeability coefficient. Therefore, during shield tunneling operations involving pressurized chamber breakthroughs, higher permeation pressure and longer permeation time are advised to allow for sufficient compression of the filter cake, thus obtaining a higher-quality filter cake.

### 3.2. Impact of Coarse Particle Materials on the Air Tightness Characteristics of Filter Cakes

#### 3.2.1. Air Tightness Pressure Value of Filter Cake

The air tightness pressure value denotes the maximum air pressure that a filter cake can withstand while preserving its air tightness. This parameter is crucial for assessing the filter cake’s capacity to endure elevated air pressures. During pressurized maintenance operations, the set air tightness differential—defined as the difference between the working pressure and external water pressure—must not exceed the filter cake’s air tightness pressure value.

**Figure 6:**
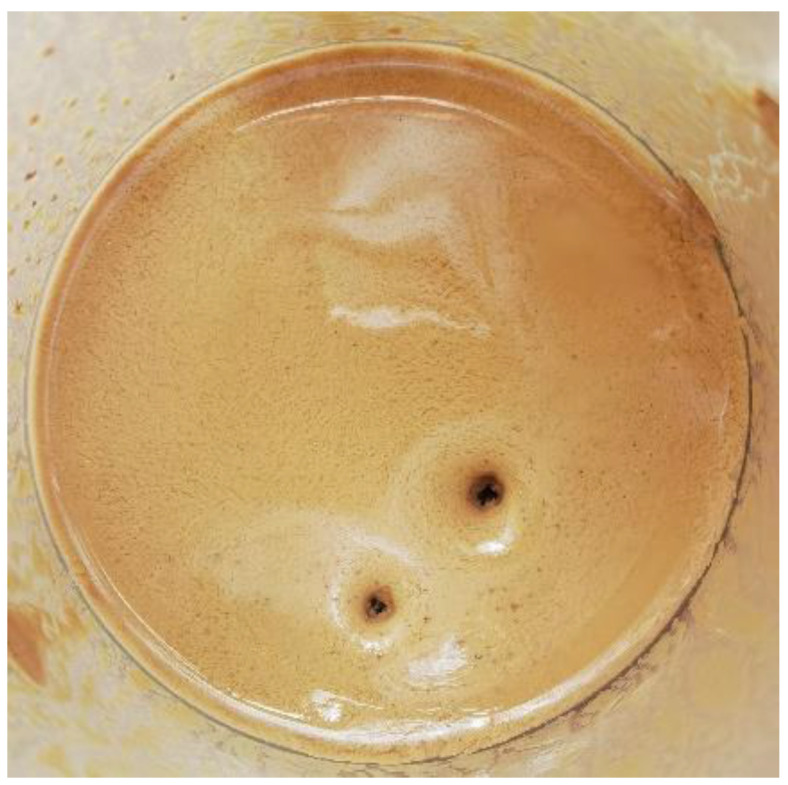
The failure morphology of filter cake in high air pressure.

In the experiment, excess slurry was drained, and incremental air pressure was applied until the filter cake failed. The failure of the filter cake under high air pressure can occur either at the moment of pressure increase at a certain pressure level or during constant pressure, manifesting as sudden instantaneous failure. The failure morphology typically appears as a circular hole breakthrough, as shown in Figure 6.

**Figure 7:**
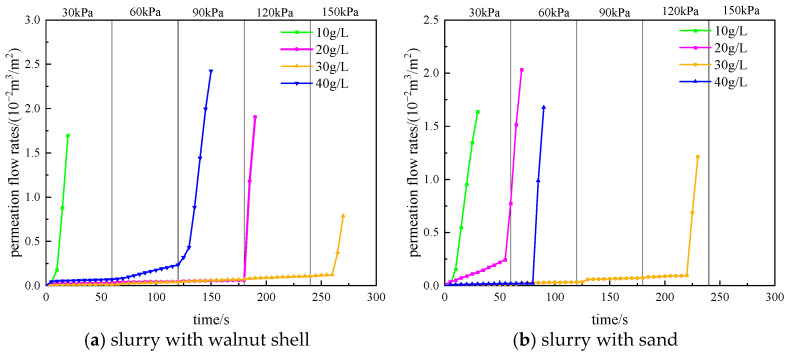
The permeation flow rate curve of the filter cake during air tightness process.

The permeation flow rate curve of the filter cake, from incremental pressure application to failure, is shown in Figure 7 (using S1 strata as an example). During each pressure level maintenance, the displacement of pore water due to air pressure causes a linear increase in the permeation flow rate of the filter cake. At the moment when the next level of air pressure load is applied, there is a slight sudden increase in permeation flow rate, primarily due to filter cake compression and drainage. After stabilization, the permeation flow rate continues to increase linearly. Once the critical point is reached, the filter cake’s ability to withstand high air pressure reaches its limit, resulting in air tightness failure.

**Figure 8:**
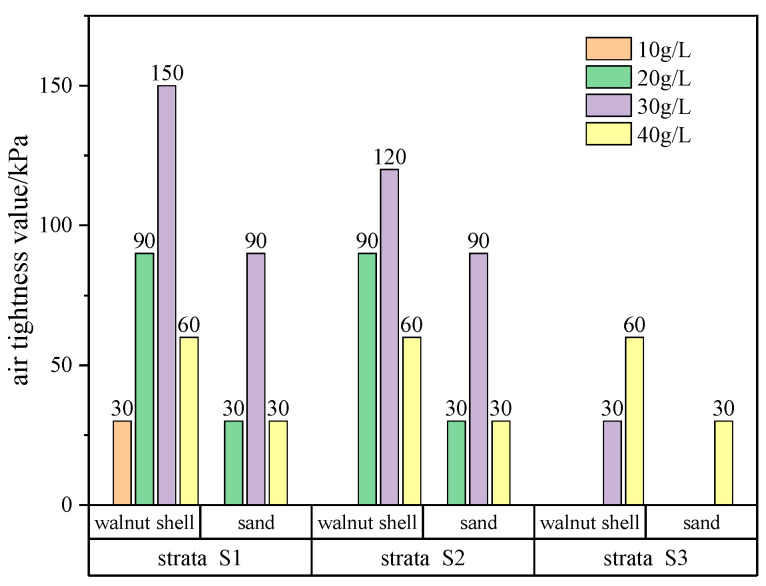
The air tightness pressure of the filter cake under varied permeability strata and coarse particle content.

The pressure level just before the failure point is considered as the air tightness pressure value of the filter cake. The air tightness pressure values of the filter cake for each group of slurries are statistically analyzed, as shown in Figure 8. As the strata permeability increases, it becomes more difficult to achieve air tightness, resulting in fewer successful air tightness outcomes and smaller air tightness pressure values for the filter cake groups. Additionally, with the enlargement of strata pores, filter cakes with higher levels of coarse particle addition gradually demonstrate advantages.

Within the same stratum, as the particle addition increases, the air tightness pressure of the filter cake initially rises and then declines. Filter cakes containing two types of particles in the S1 and S2 strata both reach their maximum air tightness pressure value at a content of 30 g/L. In the S3 stratum, only three groups of slurries successfully achieve air tightness. Filter cakes containing walnut shells reach their maximum air tightness pressure value at a content of 40 g/L, while sand-containing slurries only achieve successful air tightness at a content of 40 g/L. Comparing the two types of added particles, under the same particle addition level, the air tightness pressure value of filter cakes containing walnut shells is larger than that of filter cakes containing sand, indicating that filter cakes generated from walnut shell-containing slurries have better resistance to high air pressure.

#### 3.2.2. Air Tightness Time of Filter Cake

**Figure 9:**
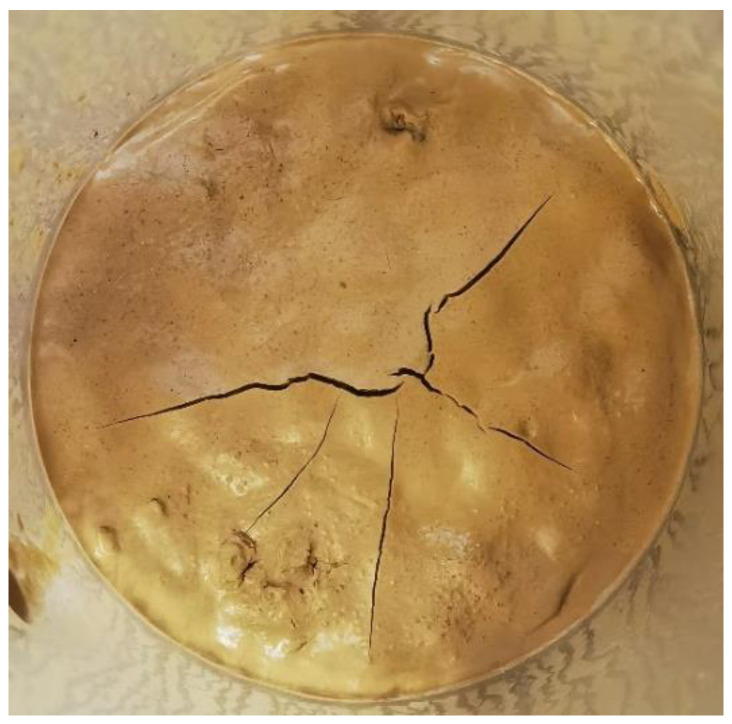
The form of failure of filter cake air tightness in constant air pressure.

The air tightness time denotes the longest duration that the filter cake can maintain its air tightness under constant air pressure. The planned maximum operating time for pressurized chamber operations must not exceed the theoretical air tightness time of the filter cake. In this experiment, a constant air pressure value of 30 kPa was applied. The failure state of the filter cake after losing air tightness under constant air pressure is illustrated in Figure 9, primarily showing dry crack failure, occasionally accompanied by multiple circular puncture holes.

**Figure 10:**
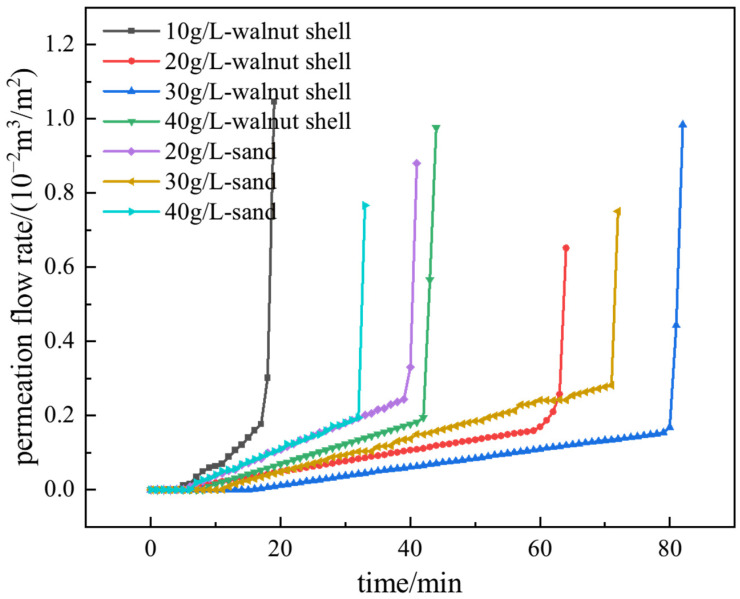
Flow volume through the filter cake under constant air pressure with varied coarse particle content.

**Figure 11:**
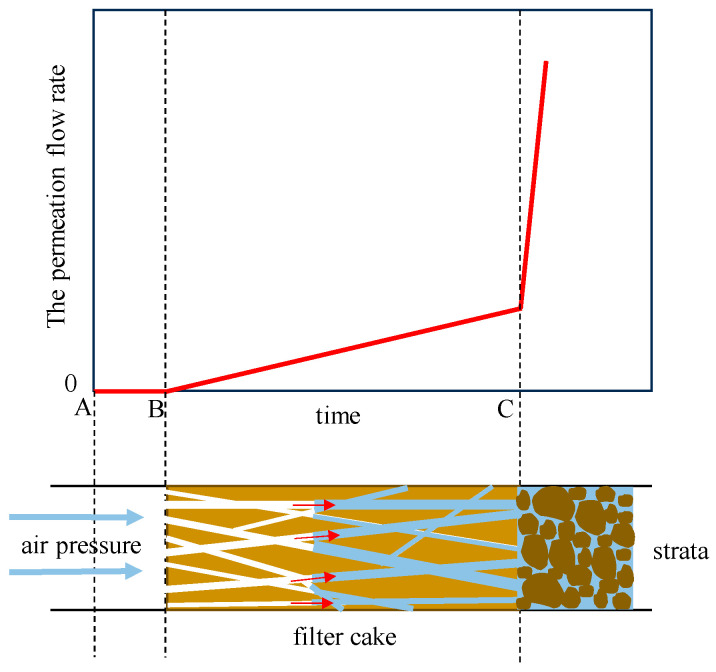
Three stages of air tightness of filter cake.

The permeation flow rate curve of the filter cake under long-term air tightness until failure under constant air pressure is shown in Figure 10 (using the S1 strata as an example). Initially, the permeation flow rate remains close to zero and gradually increases over time. At a certain moment, there is a sudden increase in permeation flow rate, indicating the air tightness failure of the filter cake. Based on the permeation flow curves monitored during the air tightness process, the process can be divided into three stages, as shown in Figure 11. The first stage is the complete air tightness stage (AB). During a short period after pressurization, the filter cake hardly generates any permeation flow rate. The second stage is the filter cake compression stage (BC). It is in this state during most of the time, and the permeation flow curve approximates a straight line. The slope of the straight line is related to the pore structure of the filter cake, the filter cake material, and its water storage capacity. The third stage is the failure stage of air tightness. When reaching point C, water in a connected pore of the filter cake is completely displaced by air, and the air tightness of the filter cake fails, resulting in a sudden increase in permeation flow rate. During the process of air displacing pore water, cracks are generated on the air pressure side of the filter cake due to dehydration. As the displacement process progresses, the cracks continuously propagate inward, resulting in dry crack failure on the upper surface of the filter cake when the air tightness of the filter cake fails.

**Figure 12:**
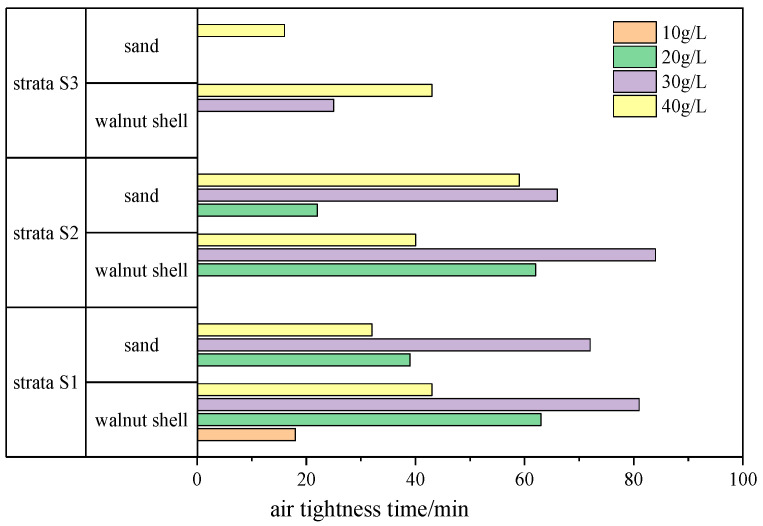
The air tightness time of the filter cake under varied permeability strata and coarse particle content.

In the experiment, the time corresponding to point C, which generates a sudden increase in flow rate, is taken as the air tightness time. The air tightness time of each group of filter cakes is statistically analyzed, as shown in Figure 12, and it follows a similar trend to the air tightness pressure values. As the permeability coefficient of the strata increases, fewer filter cake test groups reach the air tightness pressure of 30 kPa, resulting in shorter air tightness times for the slurry. In the S1 and S2 strata, filter cakes with both types of coarse particles reach the longest air tightness time at a content of 30 g/L. Moreover, filter cakes with the same content of walnut shell particles have longer air tightness times than those with sand particles. In the S3 stratum, which has the highest permeability coefficient, filter cakes with both types of particles reach the longest air tightness time when the content is 40 g/L, and their maximum air tightness time is significantly shorter compared to the previous two strata.

## 4. Discussion

Experiment results indicate that both coarse particles play a significant role in the filter cake formation of drilling slurries in highly permeable strata. Currently, the coarse particles added to the slurry are all of a single particle size, with the selection of particle size focusing more on the pore characteristics of the strata while neglecting its compatibility with the base slurry. In reality, the formation of filter cakes in highly permeable strata requires the continuous blocking formed by the base slurry, coarse particles, and the strata. The coarse particles act as a “bridge” between the strata and the fine particles such as bentonite in the slurry. If the coarse particle size is too small, they will pass through the strata pores together with the slurry, failing to serve as a skeleton for plugging and bridging. Conversely, if the coarse particle size is too large, although they can form a bridge on the surface of the strata, the skeleton of the filter cake becomes too large, still allowing fine particles in the slurry to penetrate the strata through the pores of the coarse particle skeleton, resulting in the inability to form a dense filter cake. Therefore, it is necessary to simultaneously control the maximum and minimum particle sizes of the coarse particles to achieve effective blocking.

To achieve effective plugging, the particle size of the plugging material should be larger than the pore size of the medium. Thus, the interparticle pore size can be used to determine the particle size of the plugging material. The interparticle pore range for each particle group can be obtained using the triaxial pore calculation method proposed by Herzig et al. [16]. Taking the permeation test in the S3 strata as an example, the calculated interparticle pore range for the strata particles and different sizes of coarse particles are shown in Table 5. Since the maximum particle size of the base slurry is 0.075 mm, the minimum particle group of the coarse particles should be 0.25–0.5 mm. The interparticle pore range of the strata is 0.462–0.770 mm, and the maximum particle group of the coarse particles should be 0.5–1.0 mm (standard sieve pore size). Therefore, the coarse particle range for the S3 strata to meet the filter cake forming requirements should be 0.25–1.0 mm, which is consistent with the experimental results.

Although the addition of both types of coarse particles effectively forms a filter cake, the filter cake containing walnut shell exhibited superior air tightness performance compared to the sand-containing filter cake under identical conditions. The reasons can be explained as follows. While sand-containing slurry often suffers from uneven filter cake formation due to the sedimentation effects of sand particles, walnut shell-containing slurry exhibits superior dispersion stability, facilitating the formation of a uniform and stable filter cake. As indicated by the basic properties of the slurry in Table 2, under identical conditions, the funnel viscosity of the walnut shell-containing fluid is higher than that of the sand-containing fluid, suggesting a more stable three-dimensional network structure in the former. Additionally, the ability of walnut shells to absorb water and undergo some degree of flexible deformation enhances the plugging of pore spaces in the formation, thereby promoting the formation of filter cake.

As the permeability coefficient of the strata increased, the optimal coarse particle content corresponding to the best air tightness performance of the filter cake gradually increased. The influence pattern of the content of both types of particles on air tightness performance was fundamentally consistent. Considering both the permeation flow rate of the filter cake and its air tightness performance, for high-permeability sand strata with permeability coefficients of 5.04 × 10^−3^ m/s and 5.07 × 10^−2^ m/s, it is recommended to use a coarse particle content of 30 g/L. For sand strata with a permeability coefficient of 2.30 × 10^−1^ m/s, a coarse particle content of 40 g/L is recommended.

## 5. Conclusions

To address the difficulty of filter cake formation and air tightness in high-permeability strata during the construction of slurry-water shield tunneling, comparative experiments were conducted using slurries containing two different coarse particles, walnut shells and sand, in three different permeability sand layers. The influence of coarse particles on filter cake formation and air tightness was analyzed, yielding the following main conclusions:

(1) The addition of coarse particles is essential for filter cake formation in high-permeability strata. The selection of coarse particle size should facilitate bridging effects. Controlling the maximum and minimum particle sizes based on the range of strata and coarse particle pore accumulation ensures that coarse particles can fill the strata pores while fine particles can fill the coarse particle pores.
(2) The permeation flow of the filter cake increases with increasing strata permeability, and firstly decreases and then increases with increasing particle addition. In vertical permeation, the permeation flow of walnut shell filter cake is greater than that of sand filter cake. The permeability coefficient of filter cakes with both coarse particles reaches less than 1.0 × 10^−7^ m/s, meeting the requirements of shield tunneling construction. The permeability coefficient of filter cake increases with increasing particle addition.
(3) The higher the strata permeability, the more difficult it is for the filter cake to achieve air tightness. The filter cake fails under high air pressure in the form of circular hole breakthrough damage and in the form of dry cracking damage under constant pressure. With increasing coarse particle addition, both the air tightness pressure value and air tightness time of the filter cake first increase and then decrease. Under the same conditions, filter cakes containing walnut shells exhibit a better air tightness performance than those containing sands.
(4) When performing pressurized maintenance operations with shield machines in high-permeability sand layers, using walnut shells as coarse particles instead of traditional sand can improve the air tightness performance of filter cakes. In strata with a permeability coefficient less than 5.07 × 10^−2^ m/s, a recommended walnut shell content of 30 g/L is suggested. When the strata permeability coefficient is greater than 5.07 × 10^−2^ m/s, a recommended walnut shell content of 40 g/L is suggested.

## Figures and Tables

**Table 1:** Strata parameters in experiments.

Strata Number	Dry Density/(g/cm^3^)	Porosity	Average Pore Size/(mm)	Permeability Coefficient/(m/s)
S1	1.441	0.340	0.145	5.04 × 10^−3^
S2	1.469	0.336	0.464	5.07 × 10^−2^
S3	1.510	0.330	1.002	2.30 × 10^−1^

**Table 2:** Basic parameters of slurry properties.

Slurry Number	Coarse Particle	Coarse Particle Size/mm	Added Amount of Coarse Particle/(g/L)	Added Amount of CMC/‰	24 h Funnel Viscosity/s	Specific Gravity /(g/cm^3^)
S1-0	none	—	0	1	35	1.075
S1-H-1	walnut shell	0.075–0.15	10		55	1.082
S1-H-2			20		61	1.086
S1-H-3			30		65	1.090
S1-H-4			40		68	1.092
S1-S-1	sand	0.075–0.15	10		49	1.082
S1-S-2			20		52	1.093
S1-S-3			30		57	1.100
S1-S-4			40		51	1.106
S2-0	none	—	0	2	42	1.086
S2-H-1	walnut shell	0.25–0.5	10		62	1.090
S2-H-2			20		78	1.093
S2-H-3			30		82	1.095
S2-H-4			40		84	1.097
S2-S-1	sand	0.25–0.5	10		50	1.091
S2-S-2			20		51	1.097
S2-S-3			30		52	1.100
S2-S-4			40		48	1.111
S3-0	none	—	0	3	60	1.088
S3-H-1	walnut shell	0.25–1	10		78	1.094
S3-H-2			20		77	1.096
S3-H-3			30		85	1.097
S3-H-4			40		89	1.100
S3-H-5		0.25–0.5	30		85	1.095
S3-H-6		0.5–1	30		85	1.097
S3-S-1	sand	0.25–1	10		63	1.097
S3-S-2			20		61	1.102
S3-S-3			30		63	1.103
S3-S-4			40		65	1.106
S3-S-5		0.25–0.5	30		63	1.103
S3-S-6		0.5–1	30		63	1.103

**Table 3:** Summary of slurry permeation phenomena.

Strata Number	Slurry Type	Type of Low-Permeability Zones
S1	no coarse particles	penetrating-type permeation bands
	containing 0.075–0.15 mm coarse particles	filter cake and permeation band
S2	no coarse particles	penetrating-type permeation bands
	containing 0.25–0.5 mm coarse particles	filter cake and permeation band
S3	no coarse particles	penetrating-type permeation bands
	containing 0.5–1.0 mm coarse particles	penetrating-type permeation bands

**Table 4:** Permeability coefficients of the filter cakes.

Slurry Number	Permeability Coefficient of the Filter /(10^−7^ m/s)	Slurry Number	Permeability Coefficient of the Filter/(10^−7^ m/s)	Slurry Number	Permeability Coefficient of the Filter/(10^−7^ m/s)
S1-H-1	0.92	S2-H-1	1.83	S3-H-1	1.49
S1-H-2	1.60	S2-H-2	2.04	S3-H-2	2.29
S1-H-3	2.29	S2-H-3	4.71	S3-H-3	5.25
S1-H-4	3.37	S2-H-4	7.12	S3-H-4	7.54
S1-S-1	0.79	S2-S-1	—	S3-S-1	1.07
S1-S-2	1.25	S2-S-2	1.12	S3-S-2	1.68
S1-S-3	1.56	S2-S-3	1.07	S3-S-3	2.10
S1-S-4	2.58	S2-S-4	1.91	S3-S-4	2.98

**Table 5:** Variation in pore size between particles.

Particle Size Range/mm	Interparticle Pore Range/mm
3–5	0.462–0.770
0.25–0.5	0.0385–0.0774
0.5–1	0.0774–0.154

## Data Availability

The original contributions presented in the study are included in the article, further inquiries can be directed to the corresponding author.
